# Evolutionary history and genomic consequences of polyploidization in natural populations of *Orychophragmus taibaiensis*

**DOI:** 10.1093/hr/uhaf314

**Published:** 2025-11-08

**Authors:** Qiang Lai, Zeng Wang, Changfu Jia, Xiner Qumu, Rui Wang, Zhipeng Zhao, Yao Liu, Yukang Hou, Jianquan Liu, Pär K Ingvarsson, Jing Wang

**Affiliations:** Key Laboratory for Bio-Resources and Eco-Environment of Ministry of Education, College of Life Sciences, Sichuan University, Chengdu, China; Key Laboratory for Bio-Resources and Eco-Environment of Ministry of Education, College of Life Sciences, Sichuan University, Chengdu, China; Key Laboratory for Bio-Resources and Eco-Environment of Ministry of Education, College of Life Sciences, Sichuan University, Chengdu, China; Key Laboratory for Bio-Resources and Eco-Environment of Ministry of Education, College of Life Sciences, Sichuan University, Chengdu, China; Key Laboratory for Bio-Resources and Eco-Environment of Ministry of Education, College of Life Sciences, Sichuan University, Chengdu, China; Key Laboratory for Bio-Resources and Eco-Environment of Ministry of Education, College of Life Sciences, Sichuan University, Chengdu, China; Key Laboratory for Bio-Resources and Eco-Environment of Ministry of Education, College of Life Sciences, Sichuan University, Chengdu, China; Key Laboratory for Bio-Resources and Eco-Environment of Ministry of Education, College of Life Sciences, Sichuan University, Chengdu, China; Key Laboratory for Bio-Resources and Eco-Environment of Ministry of Education, College of Life Sciences, Sichuan University, Chengdu, China; Linnean Centre for Plant Biology, Department of Plant Biology, Uppsala BioCenter, Swedish University of Agricultural Sciences, Uppsala, Sweden; Key Laboratory for Bio-Resources and Eco-Environment of Ministry of Education, College of Life Sciences, Sichuan University, Chengdu, China

## Abstract

Polyploidization has occurred throughout the tree of life and is particularly common in plants. Despite its ubiquity, our understanding of the short- and long-term effects and consequences of genome doubling in natural populations remains incomplete. In this study, we identified a novel ploidy-variable species system within the ornamental and industrial oilseed genus *Orychophragmus* (Brassicaceae), which comprises six species, including diploid and tetraploid cytotypes of *Orychophragmus taibaiensis*. By integrating population-scale genomic and transcriptomic datasets across the species in this genus, we constructed a robust phylogenetic framework and investigated the divergence and demographic history of *O. taibaiensis* in comparison to its relatives. Specifically, we characterized the geographical distribution patterns of diploids and tetraploids in natural populations of *O. taibaiensis*, confirmed the autopolyploid origin of tetraploids, and inferred their origin time relative to diploid counterparts. Our findings further revealed that, following genome doubling, tetraploids accumulated a higher genetic load of deleterious mutations, likely due to relaxed purifying selection facilitated by allelic redundancy. Additionally, genome doubling was associated with pronounced changes in gene expression patterns, with differentially expressed genes evolving under relaxed selective constraints. These results highlight that the initial masking of deleterious mutations, changes in expression regulation, and divergent efficacy of selection likely all contribute to shaping the establishment and evolutionary potential of polyploids.

## Introduction

Polyploidization, resulting from whole-genome duplication (WGD), has long been regarded as a key driver of plant speciation, adaptation to novel and extreme environments, and genetic innovation [1–4]. While allopolyploidy has received considerable attention, autopolyploidy remains markedly less studied, despite its prevalence and suitability for exploring the direct impacts of immediate genome doubling without the complications associated with hybridity [5, 6]. Autopolyploidy has been repeatedly shown to arise and establish from diploid populations, often leading to the coexistence of tetraploid and diploid populations within a single species [7, 8]. However, our understanding of the evolutionary dynamics and genomic consequences of genome doubling in these populations remains limited [9, 10]. For example, it is unclear to what extent the additional chromosomal copies resulting from WGD can mask deleterious mutations, thereby influencing the genetic load [11]. Furthermore, the degree to which genome duplication alters the efficiency of selection processes in tetraploid populations compared to their diploid progenitors remains largely unexplored [12]. Polyploidy is also frequently associated with morphological and ecological changes, but the extent to which autopolyploidy reshapes gene expression patterns and the functional roles of differentially expressed genes remains an open and intriguing question [13].

Species diversification within the Brassicaceae family is intricately linked to repeated cycles of WGDs, with polyploidy being a common feature across many lineages [14, 15]. Notably, the ancestors of the *Orychophragmus* genus underwent a unique WGD event [16], followed by rediploidization and speciation, resulting in a genome size of ~1.3 Gb—larger than that of other Brassicaceae species [17, 18]. Recently, *Orychophragmus* species have garnered attention as early-flowering ornamental plants and as promising industrial oilseed crops, owing to their high dihydroxy fatty acids content. This unique trait provides superior lubrication properties and ensures broad adaptability to diverse environmental conditions [19–22]. Moreover, their close evolutionary relationship with *Brassica* species—renowned for their ease of hybridization [23, 24]—makes *Orychophragmus* species valuable germplasm resources for *Brassica* genetics and breeding.

**Figure 1:**
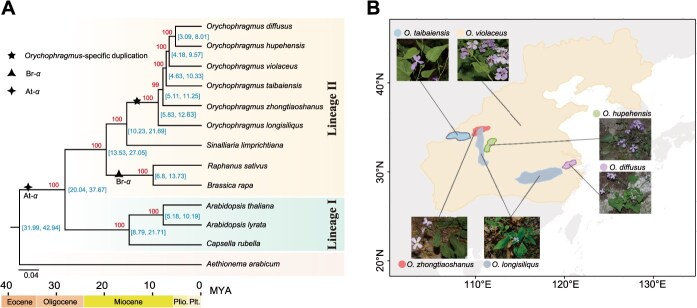
**Phylogenetic relationships and geographical distribution of**  ***Orychophragmus*****.** (A) Phylogenetic inference and divergence time estimation for six *Orychophragmus* species alongside other cruciferous species. Blue numbers (at the nodes) indicate estimated divergence times (95% highest posterior density, in million years ago [MYA]), while red numbers (above the branches) represent bootstrap support values. The black star denotes the *Orychophragmus*-specific WGD event, the black triangle marks the *Brassica*-specific triplication event, and the black tetragon represents the At-α WGD. (B) Geographical distribution and morphological characteristics of the six species within the genus *Orychophragmus*.

Despite their potential importance, the phylogenetic relationships within the *Orychophragmus* genus remain poorly understood. Currently, six species are recognized in this genus: *O. violaceus*, *O. longisiliqus*, *O. zhongtiaoshanus*, *O. taibaiensis*, *O. hupehensis*, and *O. diffusus* [25–27]. These species display significant morphological variation and inhabit diverse environments across East Asia (Fig. 1, Fig. S1). However, previous analyses have relied on a limited number of nuclear and chloroplast genes, resulting in ongoing debates about the genus’ evolutionary history and relationships. To address these uncertainties, it is essential to incorporate broader genomic datasets and advanced methodologies to construct a more robust phylogeny, with particular attention to the potential effects of incomplete lineage sorting and introgression.

**Figure 2:**
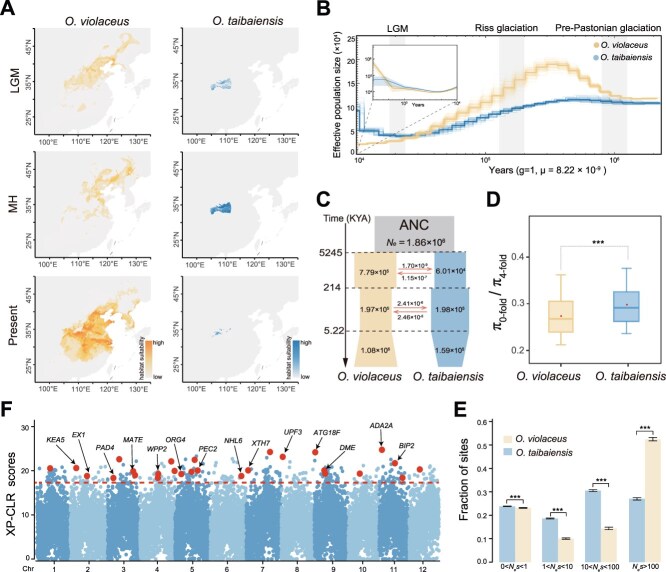
**Demographic histories and divergent selection between**  ***O. violaceus***  **and**  ***O. taibaiensis*****.** (A) Species distribution modeling under the LGM, MH, and present climate conditions. (B) Demographic history inferred using the PSMC (outer) and MSMC (inner, see Fig. S5 for details) model. Gray vertical bars indicate the LGM, Riss glaciation, and pre-Pastonian glaciation periods. Bold lines represent dynamic changes in effective population size (*N*_e_), while faint lines show 100 bootstrap replicates, ensuring robustness. (C) The best fit demographic model inferred using *fastsimcoal2*. Each block represents a current or ancestral population, with arrows indicating gene flow after divergence (per-generation migration rates). The timing of historical events is shown in KYA. (D) Ratio of nucleotide diversity at 0-fold sites relative to 4-fold sites. (E) DFE in bins of *N*_e_s for new 0-fold nonsynonymous mutations for *O. violaceus* and *O. taibaiensis*. Error bars indicate 95% CIs based on 1,000 bootstrap replicates. (F) Selective sweep analysis based on XP-CLR scores along chromosomes. The top 1% of scores, above the red dashed horizontal line (within the coordinate), are considered candidate selective regions. Red circles highlight (large, bold) representative candidate genes located within these regions, with black arrows indicating their names. Asterisks indicate statistical significance from the Wilcoxon test (two-tailed) (NS > 0.05, ^*^P < 0.05, ^**^P < 0.01, ^***^P < 0.001).

Notably, the *Orychophragmus* genus includes both widespread and regionally distributed species. *O. violaceus*, the most widely distributed species in the genus, is commonly cultivated as an ornamental plant and is known for its small purple flowers that typically bloom in early spring. In contrast, *O. taibaiensis* is endemic to the mountainous regions of the Taibai Mountains in northwest China (Fig. 2B). The contrasting distribution ranges and ecological divergence between the mountainous endemic species (*O. taibaiensis*) and the widely distributed species (*O. violaceus*) make them ideal candidates for investigating and comparing their evolutionary and demographic histories, particularly in relation to the potential accumulation of genetic load differences between species. Additionally, *O. taibaiensis* has been reported to consist of both diploid (2*n* = 2*x* = 24) and tetraploid (2*n* = 4*x* = 48) plants [28], although the mode of origin of the polyploids remains unclear. As such, it also serves as an excellent model for studying the immediate evolutionary and genomic consequences of polyploidization. A direct comparison between tetraploid populations and their diploid progenitors could provide valuable insights into how polyploidization influences both the short-term adaptive responses and long-term evolutionary potential of these populations [29, 30].

In this study, we integrated whole-genome sequencing and transcriptomic datasets to explore the phylogenetic relationships and divergence order of the six species within the genus *Orychophragmus*. We then examined the demographic and divergence history of the widespread species *O. violaceus* and the alpine endemic *O. taibaiensis*, comparing selection efficacy and the deleterious mutation load between the two species. Given that *O. taibaiensis* includes both diploid and tetraploid forms, we characterized the geographical distribution patterns of these cytotypes through extensive field investigations and karyotype analyses, while also investigating the origin of the tetraploid forms. Finally, we explored the genetic and gene expression changes associated with polyploidization in *O. taibaiensis* populations to gain deeper insights into the evolutionary consequences of WGD and its potential short- and long-term selective effects on the ecology and evolution of these populations.

## Results

### Phylogenetic and evolutionary relationships among species in the genus *Orychophragmus*

To investigate the phylogenetic relationships within the genus *Orychophragmus*, we incorporated seven additional species from the Brassicaceae family and utilized 514 single-copy genes to construct phylogenetic trees using both concatenated and coalescent approaches. Phylogenetic analysis based on single-copy genes revealed the monophyly of *Orychophragmus*, with its closest relative being the genus *Sinalliaria* (Fig. 1A). According to MCMCTree, the divergence between *Orychophragmus* and its closest relative *Sinalliaria* was estimated to be 15.43 million years ago (MYA, 95% highest posterior density: 10.23–21.69 MYA). Within the clade *Orychophragmus*, the concatenated analysis produced a strongly supported topology of outgroups (*O. longisiliqus*, (*O. zhongtiaoshanus*, (*O. taibaiensis*, (*O. violaceus*, (*O. diffusus*, *O. hupehensis*))))) (Fig. 1A). This topology was also supported by the ASTRAL coalescent approach (Fig. S2A), although some branches exhibited weak support values.

To further explore this, we used DensiTree as a visualization tool to display and quantify the concordance and discordance between individual gene trees and the species tree, which revealed significant inconsistencies between many individual gene trees and the species tree within the *Orychophragmus* clade (Fig. S2B). To further validate the phylogenetic relationship within the genus *Orychophragmus*, we integrated population-level resequencing and transcriptomic datasets, using *Sinalliaria* as the outgroup to construct phylogenies. The phylogenetic relationships within *Orychophragmus*, inferred using both maximum likelihood (ML) and neighbor-joining (NJ) methods (Fig. S3), were consistent with those derived from single-copy gene analyses (Fig. 1A, Fig. S2).

### Demographic histories and divergent selection between *O. violaceus* and *O. taibaiensis*

Given the unique features of *O. taibaiensis*—the only species in the genus reported to consist of both diploids and tetraploids as well as being the endemic species found at the highest altitudes within the genus (Fig. S4)—we specifically explored and compared the demographic histories of this species with the closely related widespread species *O. violaceus*. To achieve this, we first applied species distribution models (SDMs) to reconstruct the suitable habitats of *O. violaceus* and *O. taibaiensis* across three evolutionary periods: the present, the Middle Holocene (MH, ~6 ka), and the Last Glacial Maximum (LGM, ~21–18 ka) (see Methods for details). Our analysis revealed that the suitable habitat of *O. violaceus* expanded significantly from the LGM and MH to the present. In contrast, the suitable habitat of *O. taibaiensis* remained relatively stable, being primarily confined to the Qinling–Daba mountain regions (Fig. 2A). Furthermore, over time, its range became increasingly restricted to the Taibai Mountain region, from the LGM and MH to the present.

To assess the long-term effective population size (*N*_e_) dynamics of these two species, we applied the pairwise sequential Markovian coalescent (PSMC) method [31]. The results revealed that both species experienced a reduction in *N*_e_ from the Riss glaciation to the LGM (Fig. 2B). Consistent with the broader distribution range of *O. violaceus* predicted by the SDMs, the *N*_e_ of *O. violaceus* was generally much larger than that of *O. taibaiensis* (Fig. 2B). Since PSMC can only estimate population dynamics up to ~10,000 years ago, we also employed Multiple Sequentially Markovian Coalescent approach (MSMC2) to focus specifically on the population histories of these two species over the latest 10,000 years. The results indicated that the *O. violaceus* population underwent a more significant recovery following LGM when compared to *O. taibaiensis* (Fig. 2B; Fig. S5).

To further infer the divergence history of the two species, we employed a coalescent simulation-based approach using *fastsimcoal*2 [32, 33]. Twelve models were evaluated (Fig. S6), differing in the presence or absence of postdivergence gene flow and changes in population size following species divergence. The best fitting model (Model 12 in Fig. S6; Table S2) suggested that *O. violaceus* and *O. taibaiensis* diverged ~5.25 MYA (95% confidence interval (CI) = 1.40–7.33 MYA) (Fig. 2C; Table S3), consistent with the divergence time estimated from phylogenetic analysis (Fig. 1A). Furthermore, the model revealed divergent patterns of changes in effective population sizes and asymmetric gene flow between the species following their divergence, although no gene flow has occurred between the two species within the recent 5.22 thousand years ago (KYA) (95% CI = 5.02–5.79 KYA) (Fig. 2C, Table S2). Additionally, the model inferred that *O. taibaiensis* experienced a recent population reduction (*N_e_* = 1.59 × 10^5^, [1.45 × 10^5^–1.99 × 10^5^]), in contrast to *O. violaceus*, which underwent population expansion in both effective population size (*N_e_* = 1.08 × 10^6^ [8.45 × 10^5^–1.29 × 10^6^]) and distribution range (Fig. 2A and C).

To further compare the likely influence of different demographic histories on the efficacy of selection and the accumulation of mutational load between the two species, we estimated the ratio of nucleotide diversity at 0- to 4-fold degenerate sites. Consistent with the expectation that species with smaller population sizes have a reduced efficacy of selection to purge deleterious mutations at 0-fold sites [34], we observed that the ratio of 0- to 4-fold nucleotide diversity was significantly elevated in *O. taibaiensis* compared to *O. violaceus* (Fig. 2D). Similarly, the distribution of fitness effects (DFE) analysis revealed that *O. taibaiensis* exhibited weaker purifying selection against strongly deleterious nonsynonymous mutations (*N_e_s* > 100) compared to *O. violaceus* (Fig. 2E). Moreover, given that *O. taibaiensis* contains both diploid and tetraploid cytotypes, we reanalyzed and compared the population history divergence, genetic load, and DFE solely between *O. violaceus* and diploid *O. taibaiensis*. These results were highly consistent with the broader analysis, further supporting the conclusion that *O. taibaiensis* exhibits reduced selection efficacy and a higher mutational load compared to *O. violaceus* (Fig. S7B).

Lastly, we applied and calculated XP-CLR statistics to identify potential divergent selection regions between the two species. In total, we detected 2,397 outlier windows in the top 1% of XP-CLR scores (Fig. 2F). Further gene ontology (GO) enrichment analyses of genes within the candidate selective regions revealed significant enrichment in GO terms such as ‘cellular macromolecule metabolic process’ and ‘response to reactive oxygen species’ (Fig. S8A; Table S4), suggesting their association with divergent adaptation to different altitudinal environments between the two species. Many genes known to be involved in stress and defense responses were identified within these regions. For example, the *Arabidopsis* orthologous gene *PAD4*, which plays a critical role in salicylic acid signaling and resistance gene-mediated plant disease resistance [35, 36], was found within these regions.

In addition to the significant signals of XP-CLR, we observed substantially increased interspecies genetic divergence (*F*_ST_) and reduced intraspecies Tajima’s *D* values (Figs S8B and S9), providing strong evidence of divergent selection within these genic regions. Similarly, the *Arabidopsis* orthologous gene *PEC2*, which is involved in responding to environmental stimuli such as jasmonic acid, light, and wounding [37], was also identified, highlighting its role in stress responses and adaptive signaling pathways. We found substantially increased *F*_ST_ and reduced Tajima’s *D* values in both species around these genic regions (Fig. S8B). Similar patterns were observed for other genes related to fundamental processes crucial to plant growth, development, and epigenetic regulation, such as genes *DME* [38] and *XTH7* [39] (Fig. S8D and E). To evaluate the influence of the two cytotypes of *O. taibaiensis* on divergent selection inference, we performed XP-CLR and *F*_ST_ analyses separately using only diploid *O. taibaiensis* and combined cytotypes. The results showed high correlations (Fig. S10), indicating that ploidy differences likely had minimal impact on the overall findings. However, we acknowledge the limitations of small population sizes in this study. Future studies with broader sampling and sequencing may uncover additional insights into the genomic regions and genes driving speciation and ecological adaptation between the two species.

### Cytotype diversity and distribution in natural populations of *O. taibaiensis*

**Figure 3:**
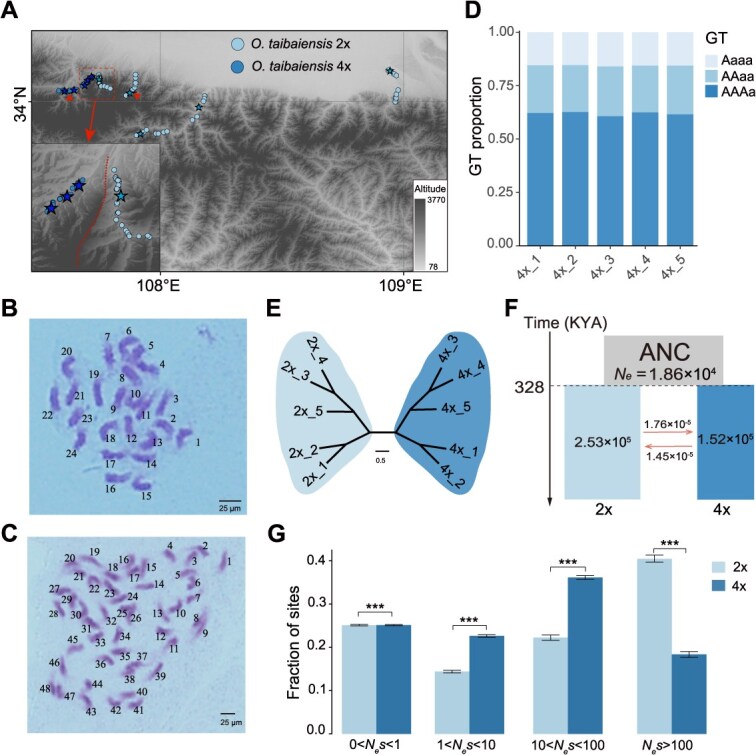
**Comparison of the geographic distribution, divergence history, genetic load, and selection efficiency of diploid and tetraploid**  ***O. taibaiensis*****.** (A) Geographic distribution of diploid and tetraploid *O. taibaiensis*, with stars representing the sequenced individuals mentioned in the text. Red triangles indicate the seed collection sites used for gene expression analysis across ploidy levels. (B) Karyotype of diploid *O. taibaiensis*. (C) Karyotype of tetraploid *O. taibaiensis*. (D) Relative proportions of various genotypes within the five whole-genome resequenced tetraploid *O. taibaiensis* individuals. (E) Phylogenetic relationships among the five resequenced diploid and tetraploid *O. taibaiensis*. (F) Population historical dynamics and divergence time estimation between diploid and tetraploid *O. taibaiensis*, based on *fastsimcoal2*. (G) DFE in bins of *N*_e_s for new 0-fold nonsynonymous mutations for diploid and tetraploid *O. taibaiensis*. Error bars represent 95% CIs based on 1,000 bootstrap replicates. Asterisks indicate the level of significance in the Wilcoxon test (two-tailed) (NS > 0.05, ^*^P < 0.05, ^**^P < 0.01, ^***^P < 0.001).

Although *O. taibaiensis* was previously reported to have both diploid and tetraploid cytotypes locally distributed in the Taibai Mountains of central China [40, 41], the spatial overlap and distribution patterns of populations with different ploidy levels remain unclear. To address this, we determined the ploidy levels of *O. taibaiensis* in a relatively large sample set of 94 individuals from various locations (Table S5). Chromosome number determination confirmed the findings of earlier studies [41] and revealed variation in chromosome size. Specifically, we identified two ploidy levels, with chromosome counts of 2n = 24 for diploids (Fig. 3B) and 2*n* = 48 for tetraploids (Fig. 3C). Cytotype distribution analysis indicated that individuals with different ploidy levels were primarily separated by local mountain barriers within relatively limited geographical ranges (Fig. 3A).

Field observations revealed no noticeable differences in landscape or morphology between the two cytotypes (Fig. S11A and B). However, under controlled conditions, tetraploid plants exhibited significantly larger organ sizes, such as increased leaf dimensions, compared to diploids (Fig. S11C and D). To further investigate whether there was environmental differentiation among cytotypes, we performed a principal component analysis (PCA) using five uncorrelated environmental variables derived from 19 bioclimatic factors (bio1-bio19) and elevation (Fig. S12A  Tables S6 and S7). The PCA results demonstrated subtle niche differentiation between cytotypes along environmental and climatic gradients (Fig. S12B). Specifically, tetraploids were associated with habitats characterized by higher maximum temperatures during the warmest month (Bio5), greater isothermality (Bio3), and lower precipitation during the driest quarter (Bio17) compared to diploids (Fig. S10C). These findings suggest that tetraploids are more likely to inhabit slightly drier and warmer environments relative to diploids of *O. taibaiensis*.

Next, we selected five diploid and five tetraploid individuals of *O. taibaiensis* for high-depth whole-genome resequencing. We first employed nQuire [42], a statistical method designed to determine the most plausible ploidy model based on the distribution of base frequencies in the sequencing data. The results from nQuire confirmed the diploid and tetraploid forms of *O. taibaiensis* that we had previously identified (Fig. S13). Specifically, the read depth density distribution of the three alleles (alternative, reference, and both) in diploid individuals was close to 1:1:2 (Fig. S14), while in tetraploid individuals, it was ~1:3:4 (Fig. S14).

To differentiate between autopolyploidization and allopolyploidization, we utilized Smudgeplots [43] to visualize the expected allele ratio patterns. In tetraploids, the AAAB pattern was more prominent than AABB (Fig. S15), consistent with the expectation of an autopolyploid origin. Furthermore, genome-wide heterozygosity analysis using GenomeScope v.2.0 revealed a distribution of allele ratios, with aaab (3.52%) being more prevalent than aabb (1.96%) (Fig. S16A), further supporting an autotetraploid origin. It is important to note that accurate polyploid inference using *k*-mer-based methods such as Smudgeplots and GenomeScope requires relatively high sequencing coverage. Thus, we restricted these analyses to polyploids with coverage exceeding 60×, all of which supported an autopolyploid origin. Lastly, we recalled SNPs based on the tetraploid model for each of the five resequenced tetraploids and calculated the proportions of various genotypes. The results indicated that the AAAa genotype was significantly more prevalent than AAaa across the entire genome (Fig. 3D; Fig. S16B–F), reinforcing the hypothesis of autotetraploidy in the polyploid *O. taibaiensis*.

### Origin history of autotetraploids and the evolutionary consequences of short-term polyploidization in *O. taibaiensis*

To investigate the origin and genetic differentiation between diploid and autotetraploid *O. taibaiensis*, we constructed an unrooted tree using 10 highly deep-sequenced individuals from both ploidy levels. The analysis revealed that the individuals formed two distinct clusters, albeit with short genetic distances and low divergence (Fig. 3E). Using *fastsimcoal2*, we identified the gene flow model as the best fit for the divergence history and estimated that diploids and tetraploids diverged ~328 KYA (95% CI = 326–468 KYA). The contemporary *N*_e_ of diploids was estimated to be 2.53 × 10^5^ (95% CI = 2.40 × 10^5^–3.61 × 10^5^), which is slightly larger than that of autotetraploids, estimated at 1.52 × 10^5^ (95% CI = 1.45 × 10^5^–2.06 × 10^5^) (Fig. 3F, Fig. S18A and B; Table S8).

To further explore the effects of ploidy on the efficacy of purifying selection, we evaluated and compared the DFE between diploids and tetraploids. The results showed that diploids exhibited a significantly higher proportion of loci under strong purifying selection (*N*_e_s > 100) compared to autotetraploids. This finding suggests relaxed purifying selection acting on nonsynonymous sites in the tetraploid population compared to the diploid population (Fig. 3G). These results remained robust when we randomly subsampled two out of four alleles per site from the genotypes of tetraploid population to account for potential biases introduced by genotype calling (Fig. S17). However, it is important to note that such biases cannot be fully resolved, as a diploid model was assumed for DFE estimation. Additionally, we calculated the ratio of overall 0- to 4-fold nucleotide diversity (Fig. S18C–I) and found that the ratio was significantly higher in autotetraploids compared to diploids. These results further suggest that the tetraploid population has accumulated a higher proportion of deleterious mutations, which aligns with the findings from *fastsimcoal2* and DFE analysis, showing that the tetraploid population, with higher genetic load, exhibits a smaller effective population size and lower purifying selection efficiency.

### Gene expression changes and associated signatures of selection upon polyploidization in *O. taibaiensis*

To explore transcriptional changes and responses to WGD, we examined gene expression differences using RNA-seq data from diploids and autotetraploids of *O. taibaiensis* in both leaf and root tissues (Table S9). PCA clearly distinguished samples based on tissue type and cytotype (Fig. S19A). To specifically investigate gene expression changes associated with polyploidization, we identified differentially expressed genes (DEGs) between diploids and autotetraploids. While the majority of genes showed no significant change in expression (NDEs), we identified 3,254 and 3,422 DEGs in leaf and root tissues, respectively (Fig. S19B), with 1,910 DEGs shared between the two tissues.

**Figure 4:**
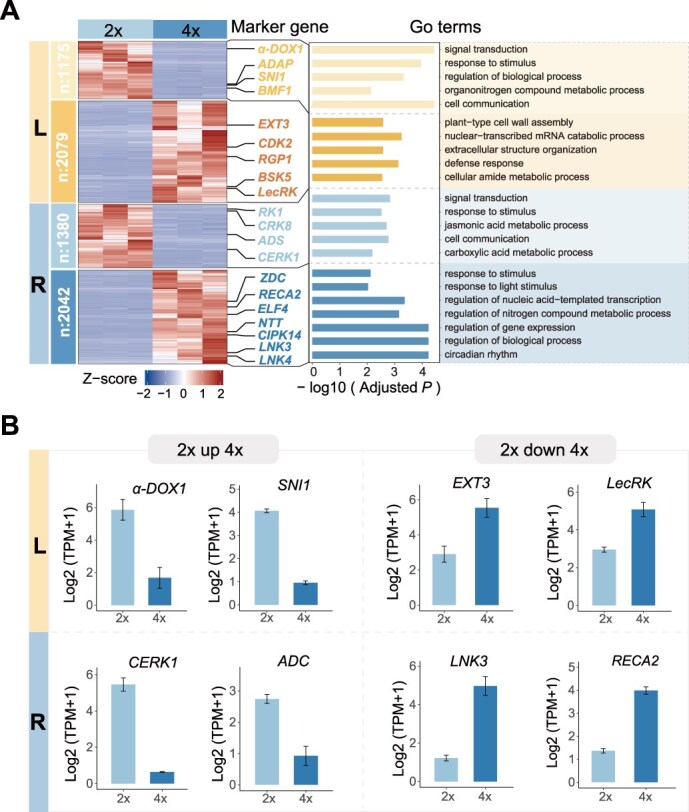
**Differential expression analysis between diploid and tetraploid**  ***O. taibaiensis*****.** (A) Hierarchical clustering of gene expression in diploid and autotetraploid *O. taibaiensis* for leaves (upper, L) and roots (lower, R), highlighting key representative GO terms enriched among DEGs within each cluster. Multiple representative DEGs from each cluster are shown. (B) Examples of expression level comparisons between diploid and tetraploid *O. taibaiensis* for selected representative DEGs identified in (A). Bar heights represent mean expression values from three biological replicates, with error bars indicating ±SD calculated across replicates.

GO enrichment analysis of these DEGs revealed that genes differentially expressed between diploids and tetraploids are highly enriched in terms related to signal transduction, cell communication, defense response, regulation of gene expression, and circadian rhythm (Fig. 4A, Table S10). For instance, genes downregulated in tetraploids, such as *SNI1* and *CERK1*, are involved in gene transcription, DNA recombination, and defense signaling (Fig. 4B). Notably, *SNI1* in *Arabidopsis* plays a crucial role in preventing errors during meiotic recombination in plants [44, 45]. Similarly, *α-DOX1* and *ADC* are key players in metabolic processes, with *α-DOX1* participating in fatty acid alpha-oxidation and *ADC* being involved in polyamine biosynthesis, both of which are essential for maintaining cellular integrity and stress responses [46, 47] (Fig. 4B). The significantly reduced expression of these genes in tetraploids may be attributed to genomic instability and the regulatory complexity caused by gene dosage effects in tetraploids. Conversely, in autotetraploid plants, the upregulated genes are primarily involved in functions critical for maintaining genomic stability and responding to environmental stresses. For example, *EXT3* is essential for plant cell wall organization, while *RECA2* is involved in DNA repair [48–50]. Additionally, *LecRK* and *LNK3* have been reported to play roles in cellular responses to salicylic acid, defense against pathogens, and the regulation of circadian rhythms [51, 52]. These findings may reflect a broader theme of adaptation and stress response, highlighting the ability of tetraploids to cope with environmental challenges (Fig. 4A and B).

**Figure 5:**
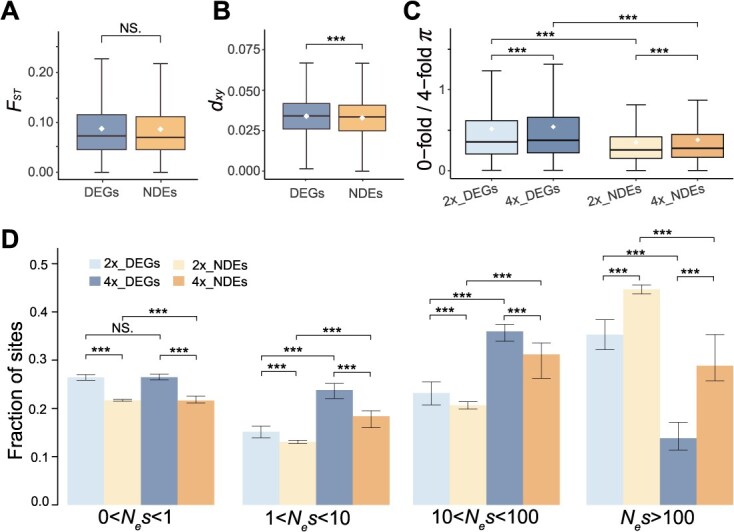
**Evolutionary and selection consequences of gene expression changes following autopolyploidization in**  ***O. taibaiensis*****.** Comparison of genetic divergence of *F*_ST_ (A), *d*_xy_ (B) between DEGs (left, in blue) and NDEs (right, in yellow) across samples with different ploidy levels. (C) Comparison of the ratio of 0- to 4-fold genetic diversity, used as a measure of selection efficiency, between DEGs and NDEs in diploid (light color) and tetraploid (dark color) populations in *O. taibaiensis*. (D) DFE for DEGs and NDEs in diploid (light color) and tetraploid (dark color) populations in *O. taibaiensis*. Errors bars represent 95% CI based on 1,000 bootstrap replicates. Asterisks denote significance levels in the Wilcoxon test (two-tailed) (NS > 0.05, ^*^P < 0.05, ^**^P < 0.01, ^***^P < 0.001).

To further investigate the association between expression divergence and sequence divergence following genome doubling in *O. taibaiensis*, we compared both the relative (*F*_ST_) and absolute (*d*_xy_) genetic divergence between the two cytotypes using whole-genome resequencing data from five diploids and five tetraploids for DEGs and NDEs. We observed that DEGs exhibited significantly higher *d*_xy_ compared to NDEs, while no significant differences were observed for *F*_ST_. These findings suggest that genes differentially expressed between diploids and tetraploids have accumulated a significantly higher average number of nucleotide differences between ploidies, while nucleotide diversity within each ploidy type remained relatively stable (Fig. 5A and B). Additionally, we compared the ratio of nucleotide diversity at 0- to 4-fold degenerate sites (π_0fold_/π_4fold_) between DEGs and NDEs and found a substantially increased π_0fold_/π_4fold_ ratio within DEGs. This result aligns with expectations of a higher mutation load and reduced purifying selection efficiency for these genes (Fig. 5C). DFE analysis further reinforced these observations, revealing that DEGs showed a significantly lower proportion of novel mutations with likely highly deleterious effects compared to NDEs, implying relaxed purifying selection and reduced functional constraints on these genes (Fig. 5D). Finally, these results were robust to random subsampling of two out of four alleles per site from the genotypes of autotetraploids. In the resampling datasets, *F*_ST_ values for DEGs were found to be significantly higher than those for NDEs, although the overall trends remained consistent (Figs S20–S22). However, we acknowledge that the sample size of resequenced individuals in this study is relatively small, which limits the robustness of conclusions regarding genetic divergence and selection efficacy. Future studies with larger population-scale datasets will be necessary to provide more robust estimates.

## Discussion

Polyploidy resulting from WGD is widespread and has repeatedly occurred throughout the evolution of eukaryotes [1, 53, 54]. However, our understanding of its effects on shaping patterns of genomic variation and influencing the selection process remains limited. In this study, we examined the nonmodel plant genus *Orychophragmus*, which includes both widespread and endemic species, as well as species with variable ploidy levels. By integrating population genomics and transcriptomic datasets, we explored the evolutionary consequences of diverse population demographic histories and the inherent effects of genome doubling on the long-term evolutionary potential of populations.

Using single-copy nuclear genes and population-level SNP datasets, our phylogenomic analyses constructed a more robust species-level phylogeny for this genus compared to previous studies [26, 55]. However, potential caveats must be considered regarding the integration of sequencing data generated from various library preparation protocols, sequencing platforms, and sampling processing procedures across species. Additionally, we observed inconsistencies among individual gene trees, likely driven by introgressive hybridization between species [56, 57], which warrants further investigation in future studies.

Notably, *O. taibaiensis* is a locally endemic mountainous species that occupies the highest elevations within the genus and is the only known species to exhibit a mixed-ploidy system. Despite this, little is known about the spatial distribution of different cytotypes across landscapes or the establishment history of polyploids [6]. To address this, we first examined the divergence history of *O. taibaiensis* and the closely related, predominantly widespread species *O. violaceus* within the genus. Ecological modeling and population genomic analyses revealed that the two species have experienced divergent demographic histories following their divergence. Specifically, *O. taibaiensis* underwent a more drastic population contraction and a mild recent population size recovery, whereas *O. violaceus* maintained a more stable demographic history. These divergent demographic trajectories have resulted in differences in selection efficacy between the two species. The lower effective population size of *O. taibaiensis* has likely led to the accumulation of a greater deleterious mutation load due to reduced selection efficacy in this species [58, 59]. Additionally, we explored signals of divergent selection between the two species and identified genomic regions enriched with genes involved in responses to oxidative stress. These genes are likely crucial for *O. taibaiensis* to survive and thrive in high mountain regions, highlighting the need for further in-depth studies to uncover the adaptive mechanisms of *O. taibaiensis* in such harsh environments.

The diploid–tetraploid variation in *O. taibaiensis* provides a unique study system to investigate the origin of polyploids, the niche similarity and differentiation of ploidies across spatial scales, and the genomic and evolutionary consequences of genome doubling [5, 60]. Through the integration of extensive field surveys, karyotype analyses, and bioinformatics approaches, our results provide strong evidence supporting the autotetraploid origin model of polyploids. Close phylogenetic relationships among individuals of the two cytotypes were observed, indicating a recent divergence history. Population demographic analyses revealed that the two cytotypes diverged ~328,000 years ago, coinciding with the late Quaternary glaciation periods, during which the Taibai Mountain was identified as a glaciation center within the Qinling Mountain range [61, 62]. These repeated glacial fluctuations, along with associated climatic and environmental changes, may have facilitated the establishment of autotetraploids, as polyploidy is widely recognized to enhance survival and establishment potential under stressful and dynamic environmental conditions [1, 63].

Notably, odd-ploidy cytotypes (e.g. triploids) were not observed in the field, suggesting that ploidy levels likely act as a strong postzygotic reproductive barrier, promoting the establishment and long-term persistence and coexistence of both cytotypes in nature [8, 64]. Across spatial scales, we clearly delineated the geographic distribution of the two cytotypes, which are primarily separated by a mountain barrier. Although generally subtle environmental differentiation between the cytotypes was observed at local spatial scales, tetraploids were found to inhabit slightly drier and warmer environments compared to diploids, suggesting a potentially higher tolerance to abiotic stress. However, further studies are needed to validate this hypothesis.

To gain deeper insights into how autotetraploids evolve and are affected by selection processes, we examined and compared the distribution of fitness effects of new mutations and the genetic load, estimated as the ratio of 0- to 4-fold diversity. Our findings suggest that polysomic masking in autotetraploids, compared to diploids, likely reduces the efficacy of purifying selection. As a result, this relaxed purifying selection leads to a higher accumulation of deleterious mutations in tetraploid populations [7, 65]. In the short term, the masking of deleterious mutations and the relaxed purifying selection that follow genome doubling may facilitate the rapid establishment and niche expansion of autotetraploid populations [1, 66]. However, it remains uncertain whether the accumulation of deleterious mutations will balance against the beneficial effects of masking, ensuring the stable persistence and coexistence of the two cytotypes in the long run. Alternatively, polyploids may represent an evolutionary dead end, with one cytotype eventually replacing the other over time [29]. Moreover, nuclear volume changes arising from polyploidization can increase genome complexity and influence gene expression [67–69]. By analyzing gene expression patterns in leaf and root tissues of the two cytotypes of *O. taibaiensis*, we identified a set of DEGs between diploids and autotetraploids, while the majority of genes were not differentially expressed (NDEs). Consistent with the expected nuclear volume changes, DEGs were significantly enriched in functions related to extracellular structure organization, cell wall assembly, and cell communication [70, 71]. Additionally, genes involved in signal transduction, response to stimuli, defense responses, regulation of primary and secondary metabolic processes, and circadian rhythm were also enriched among DEGs. These findings provide a likely explanation for the frequent association of polyploidization with enhanced stress resistance and local environmental adaptation following divergence from diploid relatives [5, 72]. Strikingly, we found that genes with expression changes between ploidy cytotypes evolve under relaxed selective constraints and accumulate more sequence divergence compared to NDEs. This highlights a strong association between expression and sequence changes in response to genome doubling for these genes. Future studies are needed to uncover the potential evolutionary forces driving this association and to explore how selection and transcriptional mechanisms jointly respond to genome doubling, shaping the subsequent evolution of autopolyploids [73, 74].

Importantly, there are a few caveats that must be acknowledged. First, biases may arise because many population genetic analyses assume a diploid model of allele frequencies at mutation–selection–drift balance, which could affect and bias the estimates for autotetraploids [7, 68]. In this study, we called variants in both diploid and polyploid modes for the autotetraploids and also performed random subsampling of two alleles per site to facilitate comparisons across various datasets. While we found consistent results, we must acknowledge that we cannot completely rule out the possibility of bias. Second, our transcriptome analyses compared the relative expression levels of genes to the total transcriptome between the two cytotypes. However, we did not estimate the true transcriptome sizes due to the lack of normalization of transcripts for cell number and biomass content changes in autotetraploids [75, 76]. This means we cannot entirely exclude the possibility that an overall change in the total number of transcripts occurred following genome doubling. Nevertheless, given that the majority of genes showed balanced expression levels between cytotypes, we believe the likelihood of substantial changes in cell number and transcriptome sizes in autotetraploids is low. Finally, future studies incorporating gene expression data from additional tissues (e.g. flowers, seeds) could offer more comprehensive insights into how transcriptional mechanisms respond to genome doubling and subsequent evolution in natural populations of *O. taibaiensis*. Additionally, the future availability of genome assemblies for both diploid and tetraploid *O. taibaiensis* could enable further exploration of the relative contributions of ancient WGDs shared by all *Orychophragmus* species and the specific WGD unique to *O. taibaiensis* in driving genome evolution and functional innovation.

Altogether, our findings provide a novel empirical study system to explore the genomic consequences of genome doubling and the potential evolutionary drivers underlying the successful establishment of newly formed autotetraploid lineages in the local mountainous endemic species *O. taibaiensis*. Future work could focus on sampling and sequencing a broader range of diploids and autotetraploids to better understand the factors influencing the evolutionary potential and establishment of polyploids, as well as their long-term coexistence with their diploid ancestors.

## Materials and methods

### Taxon sampling, genomic sequencing, and genetic data collection

We collected samples from representative natural populations of *Orychophragmus* in China, comprising a total of 17 individuals, including 10 *O. taibaiensis* (five diploids and five tetraploids) from the Taibai Mountains, 1 *O. longisiliqus* and 2 *O. zhongtiaoshanus* from Shanxi and Shaanxi Province, 1 *O. hupehensis* from Hubei Province, and 3 *O. violaceus* from Henan and Shaanxi Provinces. Sampling details, including coordinates and voucher information, are provided in Table S1. Fresh young leaves were immediately dried in silica gel for DNA extraction. For the newly sequenced dataset, genomic DNA was extracted from leaf samples using the Qiagen DNeasy Plant Kit, and whole-genome paired-end reads (PE150) were generated on the BGI T7 platform.

After integrating newly sequenced genomic datasets from this study with transcriptomic datasets from previous research [55], we collected genomic and/or transcriptomic data for 52 individuals, representing all six *Orychophragmus* taxa: 7 *O. longisiliqus*, 13 *O. zhongtiaoshanus*, 10 *O. taibaiensis*, 1 *O. hupehensis*, 6 *O. diffusus*, and 14 *O. violaceus*. Additionally, we included one species of *Sinalliaria limprichtiana* as the outgroup. We integrated both resequencing and transcriptomic data for phylogenetic analysis of the *Orychophragmus* species. Subsequently, genomic data from resequencing were used to study the population evolutionary history of *O. violaceus* and *O. taibaiensis*.

### Phylogenomic construction

To construct the phylogenetic relationships among *Orychophragmus* species and their relatives, we utilized RNA-Seq reads from *O. longisiliqus* (SRR6655848), *O. diffusus* (SRR6655832), *O. violaceus* (SRR6655842), and the closely related lineage *S. limprichtiana* (SRR6441722) for *de novo* transcriptome assembly. The RNA-Seq reads were processed using fastp v.0.20.1 [77] for quality control, followed by *de novo* assembly with Trinity v.2.8.5 [78] under default parameters. The resulting assemblies were refined by retaining only the longest isoform for each gene and removing redundant sequences using CD-HIT v4.8.1 [79]. Completeness was assessed with BUSCO v.5.3.0 [80], and protein-coding regions were identified using TransDecoder v.5.26.3 [81]. An OrthoFinder v.2.5.4 [82] analysis of the four transcriptomes identified 3021 single-copy orthologous genes. Using the highest quality assembly (SRR6655848) as the reference, we employed aTRAM v.2.4.0 [83] to extract and reassemble these genes from three additional species with whole-genome resequencing data (*O. zhongtiaoshanus*: LaiQ184P1, *O. taibaiensis*: LaiQ179P10, *O. hupehensis*: LaiQ197P1). After integrating the assembled sequences with protein data from six additional Brassicaceae species (*Aethionema arabicum*: PRJNA202984, *Capsella rubella*: GCA_000375325, *Arabidopsis lyrata*: GCA_000004255, *Arabidopsis thaliana*: GCA_000001735, *Raphanus sativus*: GCA_010725405, and *Brassica rapa*: GCA_000309985), OrthoFinder identified 514 conserved single-copy genes (>300 bp) suitable for phylogenetic reconstruction across all species.

To construct the phylogenetic relationships, protein sequences were aligned with MAFFT v7.475 [84] and converted to codon-based nucleotide alignments using PAL2NAL [85]. After removing spurious sequences and poorly aligned regions with trimAl v1.4.rev22 [86], the remaining alignments were combined into a supergene matrix for phylogenetic construction using RAxML v8.2.8 [87] under the GTRCAT model. To estimate divergence times between species or clades, we employed MCMCTree from the PAML v4.10.0 package [88, 89], incorporating fossil calibration points obtained from TimeTree [90]. Additionally, we performed coalescent-based species tree inference using ASTRAL v.5.15.5 [91] to account for potential discordance among gene trees, with the results visualized through DensiTree v2.2.7 [92]. to examine topological conflicts between individual gene trees and the consensus species tree.

We further used population genomic data to confirm the interspecific relationship within *Orychophragmus.* Whole-genome resequencing data and transcriptome data were combined to extract variant sites. We processed quality control of raw genomic resequencing and transcriptome sequencing data using fastp v.0.20.1 [77] with stringent quality filtering parameters (-3 20 -5 20 -M 20 -l 36) to ensure data quality. The filtered genomic resequencing reads were then aligned to our newly assembled *O. violaceus* [93] genome using the BWA-MEM algorithm of bwa v.0.7.17 [94] with default settings, whereas the filtered transcriptome sequencing high-quality reads were mapped using HISAT2 v. 2.2.1 [95]. All alignments were processed using SAMtools v.1.9 [96] for sorting and Picard v.2.18.21 for polymerase chain reaction (PCR) duplicate marking (http://broadinstitute.github.io/picard/). Genetic variants (SNP calling) were identified using the Genome Analysis Toolkit (GATK) v.4.2.5.0 [97], including HaplotypeCaller, CombineGVCFs, and GenotypeGVCFs modules with the ‘EMIT_ALL_SITES’ parameter to retain both variant and invariant sites. After combining the GVCF files from the resequencing and transcriptome datasets, the GenotypeGVCFs tool was applied to genotype the variants, which was followed by applying a strict set of filtering criteria: first, we masked non-uniquely mappable regions using Heng Li’s SNPable tool (http://lh3lh3.users.sourceforge.net/snpable.shtml). The reference genome was split into 100-mer sequences and realigned (bwa aln -R 1000000 -O 3 -E 3), retaining only uniquely mapped sites (712,157,434 out of 1,285,736,775 sites). Subsequent filtering removed: (1) multiallelic SNPs (>2 alleles); (2) extreme depth variants (DP <5 or >3× average depth); (3) low-quality SNPs (QD <2.0, FS >60, MQ <20, MQRankSum < −12.5, ReadPosRankSum < −8.0); and (4) sites with >20% missing data. Finally, we identified 4- and 0-fold degenerate sites using the Degeneracy tool (https://github.com/tvkent/Degeneracy) and merged these with transcriptomic data from *S. limprichtiana* (SRR6441722) as outgroup. The final dataset contained 93,969 high-confidence SNPs for population genomic phylogenetic reconstruction.

NJ phylogenetic trees were first constructed using PLINK v.1.90 [98] with the parameter distance 1-ibs to calculate the pairwise identify-by-state (IBS) genetic distance matrix, followed by MEGA X [99] for tree construction. And for ML phylogenetic trees were constructed using IQ-TREE v.2.2.0.3 [100], where ModelFinder [101] was used to select the best fitting substitution model (-B 1000 -m MFP) with 1,000 ultrafast bootstraps.

### Demographic history analyses of *O. violaceus* and *O. taibaiensis*

To reconstruct the historical demography of the widespread species *O. violaceus* and the locally endemic species *O. taibaiensis*, SDMs [102] were developed using MAXENT v.3.4.4 [103, 104]. Based on ecological niche theory, SDMs infer a species’ potential geographic distribution by combining occurrence records with multiple environmental predictors, thereby estimating habitat suitability under various climatic scenarios. These models were applied to evaluate shifts in suitable habitat ranges across different time periods for both species. To achieve this, current native distribution records for *O. violaceus* (445 records) and *O. taibaiensis* (91 records, both cytotypes combined) were collected from the Chinese Virtual Herbarium (CVH, https://www.cvh.ac.cn/) and our field investigations.

We utilized 19 bioclimatic variables (BIO1-BIO19) at 2.5-arc-minute resolution from WorldClim (http://www.worldclim.org) [105], examining three temporal scenarios: the present (1970–2000), MH (~6,000 years ago), and LGM (~20,000 years ago). Separate species distribution models were calibrated for each species: one for *O. violaceus* using 445 occurrence records, and one for *O. taibaiensis* using 91 combined records of both cytotypes. Both models were trained under present-day climatic conditions (1970–2000) to characterize the species’ current ecological niche. The calibrated models were then projected onto the paleoclimatic reconstructions of the MH and LGM periods to infer potential suitable habitats during these historical epochs. To ensure model robustness, we excluded highly correlated variables (Pearson’s *r* ≥ 0.8). Model performance was assessed using the area under the receiver operating characteristic (ROC) curve (AUC) metric. [106], which provides reliable evaluation independent of specific thresholds or species prevalence.

To comprehensively reconstruct the demographic histories of these species, we employed a multitiered coalescent-based approach. For long-term effective population size (*N_e_*) dynamics, we implemented the PSMC method [31] with parameters ‘N25 -t15 -r5 -p “4+25×2+4+6”’, conducting 100 bootstrap replicates to assess estimation robustness. The analysis incorporated a mutation rate of 8.22 × 10^−9^ per site per generation [107] and an annual generation time assumption for temporal scaling of the results. To gain deeper insights into recent population dynamics, particularly over the past 10,000 years, we employed the MSMC2 [108]. This analysis was performed on phased whole-genome sequences that were generated by Beagle v.4.1 [109] from four individuals (representing eight haplotypes) for each species. For both species, MSMC2 was run on all possible individual configurations, and the medians and SDs of *N_e_* changes were subsequently estimated.

Subsequently, we inferred the divergence history between *O. violaceus* and *O. taibaiensis* using a coalescent simulation-based approach implemented in *fastsimcoal*2 v.27 [32, 33]. The analysis was conducted using 4-fold degenerate sites extracted from whole-genome resequencing data to construct a 2D site frequency spectrum (2D-SFS) through the easySFS pipeline (https://github.com/isaacovercast/easySFS). Twelve distinct demographic models were systematically evaluated, representing various evolutionary scenarios including strict isolation, divergence with or without gene flow, and postdivergence population size changes (Fig. S6). Each model underwent rigorous evaluation through 50 independent optimization runs, with each run performing 100,000 coalescent simulations across 40 iterative cycles to ensure robust parameter estimation. Model selection was based on maximum likelihood optimization, with additional evaluation using Akaike weights and Δlikelihood comparisons. To assess CIs, we performed an extensive bootstrapping procedure consisting of 100 bootstrap replicates, each including 50 independent optimization runs. Throughout the analysis, we maintained consistency with our previous demographic analyses by applying the same mutation rate of 8.22 × 10^−9^ mutations per site per generation [107] and assuming an annual generation time.

### Assessment of selection efficiency and divergent selection signatures between *O. violaceus* and *O. taibaiensis*

Given the differences in demographic history and effective population sizes between *O. violaceus* and *O. taibaiensis*, we compared the efficacy of natural selection and the deleterious genetic load between the two species. First, we calculated and compared the genome-wide ratio of nonsynonymous to synonymous nucleotide diversity (π_0-fold_/π_4-fold_) using pixy v1.0.4 [110] across 100-kb nonoverlapping window. Next, we estimated the DFE for newly arising nonsynonymous mutations using DFE-alpha v2.16 [111]. This analysis incorporated a demographic model with stepwise population size changes that was first fitted to the neutral SFS. The estimated demographic parameters were then used to infer both the fitness effects of deleterious mutations and the strength of purifying selection (*N_e_s*) specific to each species. To evaluate the robustness of our estimates, we performed 1,000 bootstrap replicates by resampling sites within each functional category while excluding the top and bottom 2.5% of values, generating 95% CIs for all parameters. Furthermore, to investigate genomic regions potentially involved in species divergence, we performed genome-wide scans for divergent selection between *O. violaceus* and *O. taibaiensis* using the XP-CLR method [112]. Our analysis calculated composite likelihood ratios (XP-CLR scores) across 5-kb nonoverlapping windows, with the top 1% of windows (*n* = 2,397) identified as candidate selection regions. These regions were further validated through complementary analyses of population differentiation (*F*_ST_) and Tajima’s *D* statistics to confirm signatures of selection. Finally, we performed GO enrichment analysis on the 340 genes located in these candidate regions using the topGO package (https://bioconductor.org/packages/topGO/) to characterize their functional profiles.

### Determination of diploid and tetraploid distribution across *O. taibaiensis* populations

To thoroughly characterize the geographical distribution of diploid and tetraploid cytotypes across the natural populations of *O. taibaiensis*, and based on the descriptions in the original literature of *O. taibaiensis* (Fig. 1B) [40], we conducted extensive sampling across the entire distribution area. Seeds were collected from 94 individuals across seven populations, covering the entire known distribution of *O. taibaiensis* identified in the field (Fig. 3A). Following seed germination on moist filter paper in Petri dishes at 25°C, root meristems were processed through a standardized protocol: (1) pretreatment with 0.1% colchicine solution (25°C, 3 h), (2) fixation in ethanol-acetic acid (3:1) at 4°C for 3 h, (3) hydrolysis in 1 M HCl (37°C, 45 min), and (4) staining with modified carbol-fuchsin solution (room temperature, ≥3 h). Chromosome preparations were made by squashing stained meristems, with counts determined using oil immersion microscopy (1000× magnification) and documented photographically.

To further elucidate and differentiate the origin of polyploidy in *O. taibaiensis*, we conducted a comprehensive genomic analysis to distinguish between autopolyploid and allopolyploid formation mechanisms. Based on the geographical distribution of diploid and tetraploid individuals, we selected five diploid and five tetraploid individuals (as indicated by the asterisks in Fig. 3A) for high-depth whole-genome resequencing (average depth ~52.48×), representing the key distribution locations of the two cytotypes. Initial ploidy confirmation was achieved through nQuire [42] analysis, which involved noise reduction using the ‘denoise’ algorithm followed by reference genome alignment to assess read depth distributions and allele frequencies. We also calculated the density distribution of the three allele types (reference, alternative, and both) to validate ploidy levels. Next, we integrated GenomeScope and Smudgeplots [43] to distinguish between autotetraploids and allotetraploids. This analysis involved examining genome-wide heterozygosity patterns in polyploid samples using *k*-mer frequency spectra that were generated from the resequencing data through Jellyfish v2.2.9 [113]. Finally, we reperformed variant calling for the five resequenced individuals of tetraploid *O. taibaiensis* using GATK, setting the parameter ‘–ploidy 4’. The relative proportions of different genotypes (e.g. Aaaa, AAaa, aaaA) were calculated to further verify whether the tetraploids were of autotetraploid or allotetraploid origin.

### Population genomic analysis and assessment of genetic load for diploid and tetraploid individuals of *O. taibaiensis*

To construct the phylogenetic relationships of *O. taibaiensis* at different ploidy levels, we reperformed variant calling and filtering, following the method described in Demographic histories and divergent selection between *O. violaceus* and *O. taibaiensis* section, for the 10 high-depth resequenced individuals. First, we calculated pairwise genetic distances using IBS metrics implemented in PLINK v1.90 [98] with parameter settings for distance calculation (distance 1-ibs). These distance matrices were then used to construct an unrooted NJ phylogenetic tree in MEGA X [99], revealing the genetic clustering patterns among individuals of different ploidy levels. Next, we further utilized *fastsimcoal2* [32] to infer the divergence history of the two cytotypes based on a 2D joint SFS constructed from 4-fold degenerate sites. Whole-genome resequencing data from *O. longisiliqus* and *O. zhongtiaoshanus* were included as outgroups to infer the derived states of alleles using the est-sfs software [114]. The analysis was performed under two models: one accounting for gene flow and one assuming no gene flow (see Fig. S14A and B). The detailed procedures followed those outlined in Cytotype diversity and distribution in natural populations of *O. taibaiensis* section.

We conducted a comparative analysis to evaluate how polyploidization influences the efficiency of purifying selection and the accumulation of deleterious mutations in *O. taibaiensis.* Our approach involved calculating and comparing the ratio of nonsynonymous to synonymous nucleotide diversity (π_0-fold_/π_4-fold_) and the DFE for newly arising mutations across both ploidy levels. The intensity of purifying selection (*N_e_s*) at nonsynonymous sites was estimated using DFE-alpha v2.16 [111] by fitting a stepwise population size change model to account for differences in the SFS of 0-fold nonsynonymous sites and putatively neutral 4-fold synonymous sites. To evaluate the potential effects of the diploid assumption in these analyses, we reperformed specialized variant calling for tetraploid individuals using GATK, setting the parameter ‘–ploidy 4’. For each tetraploid individual, we randomly subsampled two alleles from the four alleles per site to generate six independent subsampling datasets. These datasets were then used for associated analyses, including *fastsimcoal*2 modeling, genetic load estimation, and DFE analysis. This approach ensured robust comparison between diploid and tetraploid cytotypes while accounting for the polyploid nature of the tetraploid individuals.

### Differential gene expression analysis between the two cytotypes of *O. taibaiensis*

To investigate how polyploidy influences transcriptomic changes, seeds of diploid and tetraploid *O. taibaiensis* collected from two geographic regions (Fig. 3A red triangles) were grown under identical conditions in a growth chamber. The environment was controlled with a 16/8-h light/dark photoperiod, a constant temperature of 25°C, and a light intensity of 100 μmol m^−2^ s^−1^. After 6 months of growth, leaf and root tissues were collected from plants with similar growth characteristics for both cytotypes. Three biological replicates were collected for each ploidy level to ensure statistical reliability. Immediately after collection, plant tissues were flash-frozen in liquid nitrogen and stored at −80°C to preserve RNA integrity. Total RNA extraction was performed according to the TIANGEN RNA Kit protocol, and subsequent library preparation yielded high-quality sequencing libraries that were processed on the DNBSEQ-T7 platform using paired-end sequencing methodology.

The RNA-seq data analysis pipeline began with quality control of raw sequencing reads using fastp v0.20.1 [77] with stringent quality filtering parameters (-3 20 -5 20 -M 20 -l 36) to ensure data quality. Processed reads were then aligned to the *O. violaceus* reference genome [93] using HISAT2 v2.2.1 [95], followed by transcript quantification with StringTie v1.3.6 [115, 116] to obtain transcripts per million (TPM) values. Of the 52,812 annotated genes in the genome, we detected expression (TPM >0) for 37,607 genes across all examined tissues and ploidy levels.

For differential expression analysis, we employed DESeq2 [117] with stringent significance thresholds of |log2 fold change (FC)| ≥2 and adjusted *P*-value < 0.001 to identify significant polyploidy-associated DEGs. Genes showing minimal expression changes with |log2 fold change (FC)| <1 or lacking statistical significance (adjusted *P*-value > 0.05) were classified as NDEs. The DEGs were subsequently analyzed for expression pattern clustering using the ClusterGVis package’s mfuzz algorithm (https://github.com/junjunlab/ClusterGVis) and subjected to functional annotation through GO enrichment analysis implemented in the topGO package.

### Selection on genes exhibiting differential expression between cytotypes

To examine and compare differences in the strength and direction of natural selection on DEGs and NDEs associated with polyploidization in diploids and tetraploids of *O. taibaiensis*. We implemented a three-pronged analytical approach to compare selection patterns: firstly, we quantified pairwise relative genetic divergence (*F*_ST_) and absolute genetic divergence (*d*_xy_) measures between diploid and tetraploid cytotypes for the DEGs and NDE gene sets using PIXY v1.0.4 [110]. Next, we evaluated mutational load by computing the ratio of nonsynonymous to synonymous nucleotide diversity (π_0-fold_/π_4-fold_) between the two gene sets. Finally, we assessed the strength of selective constraint on the two gene sets by modeling the DFE. As described earlier, the strength of purifying selection (*N*_e_s) on 0-fold nonsynonymous sites was estimated for each gene set in both diploids and tetraploids, using 4-fold synonymous sites as a neutral reference, with DFE-alpha v2.16 [111]. For tetraploid individuals, six additional independent datasets, generated by randomly sampling two alleles from the four alleles, were applied in all analyses in this section.

## Supplementary Material

Web_Material_uhaf314

## Data Availability

All data needed to evaluate the conclusions in this study are present in the paper and/or the Supplementary information. The newly generated whole-genome resequencing data and transcriptome data of the samples produced in this study have been deposited in the National Genomics Data Center (https://ngdc.cncb.ac.cn) under the accession number PRJCA035915.
